# Resolvin D3 controls mouse and human TRPV1-positive neurons and preclinical progression of psoriasis

**DOI:** 10.7150/thno.52135

**Published:** 2020-10-26

**Authors:** Sang Hoon Lee, Raquel Tonello, Sang-Taek Im, Hawon Jeon, Jeongsu Park, Zachary Ford, Steve Davidson, Yong Ho Kim, Chul-Kyu Park, Temugin Berta

**Affiliations:** 1 Pain Research Center, Department of Anesthesiology, University of Cincinnati Medical Center, Cincinnati, OH, USA.; 2 Gachon Pain Center and Department of Physiology, College of Medicine, Gachon University, Incheon 21999, Republic of Korea.; 3 Department of Health Sciences and Technology, GAIHST, Gachon University, Incheon 21999, Korea, Republic of Korea.

**Keywords:** psoriasis, skin inflammation, sensory neurons, pruritus, resolvin D3, TRPV1, CGRP

## Abstract

**Rationale:** Psoriasis is a chronic inflammatory disease caused by a complex interplay between the immune and nervous systems with recurrent scaly skin plaques, thickened stratum corneum, infiltration and activation of inflammatory cells, and itch. Despite an increasing availability of immune therapies, they often have adverse effects, high costs, and dissociated effects on inflammation and itch. Activation of sensory neurons innervating the skin and TRPV1 (transient receptor potential vanilloid 1) are emerging as critical components in the pathogenesis of psoriasis, but little is known about their endogenous inhibitors. Recent studies have demonstrated that resolvins, endogenous lipid mediators derived from omega-3 fatty acids, are potent inhibitors of TRP channels and may offer new therapies for psoriasis without known adverse effects.

**Methods:** We used behavioral, electrophysiological and biochemical approaches to investigate the therapeutic effects of resolvin D3 (RvD3), a novel family member of resolvins, in a preclinical model of psoriasis consisting of repeated topical applications of imiquimod (IMQ) to murine skin, which provokes inflammatory lesions that resemble human psoriasis.

**Results:** We report that RvD3 specifically reduced TRPV1-dependent acute pain and itch in mice. Mechanistically, RvD3 inhibited capsaicin-induced TRPV1 currents in dissociated dorsal root ganglion (DRG) neurons via the N-formyl peptide receptor 2 (i.e. ALX/FPR2), a G-protein coupled receptor. Single systemic administration of RvD3 (2.8 mg/kg) reversed itch after IMQ, and repeated administration largely prevented the development of both psoriasiform itch and skin inflammation with concomitant decreased in calcitonin gene-related peptide (CGRP) expression in DRG neurons. Accordingly, specific knockdown of CGRP in DRG was sufficient to prevent both psoriasiform itch and skin inflammation similar to the effects following RvD3 administration. Finally, we elevated the translational potential of this study by showing that RvD3 significantly inhibited capsaicin-induced TRPV1 activity and CGRP release in human DRG neurons.

**Conclusions:** Our findings demonstrate a novel role for RvD3 in regulating TRPV1/CGRP in mouse and human DRG neurons and identify RvD3 and its neuronal pathways as novel therapeutic targets to treat psoriasis.

## Introduction

The interaction between the immune and nervous system is a rapidly evolving topic in various skin diseases 1-3, including psoriasis. Psoriasis affects 2-3% of the world's population, and is characterized by the presence of scaly skin plaques, thickened stratum corneum, infiltration and activation of inflammatory cells, arthritis and itch 4. The involvement of the nervous system in psoriasis is emphasized by clinical studies showing that this disease can be exacerbated by psychosocial stress 5,6, and improved by denervation of the skin 7,8. The skin is innervated by an intricate network of nociceptive sensory neurons, known as nociceptors, the primary function of which is to protect our body from the transmission of sensations such as pain and itch 9,10. Recently, preclinical studies have demonstrated how specific nociceptors and the receptors expressed in these neurons are involved in psoriasis by promoting chronic itch and skin inflammation 11-13, thus implicating these neurons as novel therapeutic targets.

Of particular interest, transient receptor potential (TRP) channels such as TRPV1, TRPA1 and TRPC4 are selectively expressed in nociceptors and emerge as major drivers of acute itch, with TRPV1 underlying histaminergic itch 14, TRPC4 contributing to histaminergic and serotonergic itch 12,15, and TRPA1 driving most other non-histaminergic itch sensations 16,17. These channels have also shown different contributions to various psoriatic symptoms, with TRPV1 and TRPC4 being detrimental and TRPA1 being beneficial for psoriasis in preclinical studies 11,12,18,19. Great pharmaceutical efforts have been devoted to the development of small-molecules targeting TRP channels, with TRPV1 antagonists yielding multiple clinical trials 20. Unfortunately, most of these antagonists have shown adverse effects such as hyperthermia and reduced heat pain threshold hampering their translation into therapeutics in humans 21.

Resolvins (RvDs) are endogenous lipid mediators generated from omega-3 polyunsaturated fatty acids (omega-3s) with neuroimmune and pro-resolution actions 22. Interestingly, RvDs significantly decrease the activity of TRP channels through G protein-coupled receptors (GPCRs) without known adverse effects 23. For instance, RvE1 blocked spontaneous pain induced by the naturally occurring TRPV1-agonist capsaicin and reduced inflammatory pain 24, whereas RvD1 blocked TRPA1 activity in small-sized neurons (i.e. putative nociceptors) from dorsal root ganglia (DRGs) and alleviated sciatica 25,26. While these resolvins have been reported to reduce psoriatic skin inflammation in a preclinical animal model 27,28, the underlying mechanisms, the therapeutic effects on itch, and potential translation into human pathology still remain unclear.

Here, we report the anti-inflammatory and anti-pruritic effects as well as underlying neuroimmune mechanisms of RvD3, a novel resolvin family member with a well-established structure and synthesis 29. RvD3 is endogenously expressed in mouse and human tissues and various studies have now demonstrated beneficial effects of RvD3 in infections, arthritis and spinal cord injury 30-32. In our study, we demonstrate that the administration of RvD3 in mice significantly reduces both skin inflammation and chronic itch in a well-characterized animal model of psoriasis. Behavioral and electrophysiological analyses reveal that RvD3 inhibits TRPV1 activity via GPCR signaling and release of the pro-inflammatory calcitonin gene-related peptide (CGRP) from DRG neurons. Most importantly, we confirm RvD3 inhibitory functions in DRG neurons from human donors, thereby increasing confidence in the relevance of targeting neuronal mechanisms and the use of RvD3 in novel therapeutic strategy to effectively and safely control psoriasis.

## Methods

### Animals and human samples

All protocols for animal experiments were approved by the Institutional Animal Care & Use Committees of the University of Cincinnati and Gachon University, and all procedures were keeping within the National Institutes of Health Guide for the Care and Use of Laboratory Animals. Adult CD-1 mice (males, 8-10 weeks, Charles River Laboratories, Wilmington, MA) were used for behavioral and biochemical studies. Young CD-1 mice (6 weeks) were used for electrophysiological studies in dissociated neurons from DRGs. All animals were housed under a 12-h light/dark cycle with food and water available ad libitum. Although no statistical power calculation was performed, sample sizes were estimated based on our previous studies for similar types of behavioral, biochemical and electrophysiological analyses 15,33,34. Animals were randomly assigned to experimental groups, and experimenters were blinded to treatment conditions. Studies involving human DRGs were performed on biospecimens from deidentified and non-diseased human donors and approved by the Institutional Review Boards at University of Cincinnati.

### Reagents

We purchased RvD3 (cat. no. 13834) from Cayman Chemical, 5% imiquimod (IMQ) cream from Perrigo (Dublin, Ireland), and control vehicle cream (Vaseline, 100% pure petroleum jelly) from Unilever (Trumbull, CT). Histamine (cat. no. H7125), serotonin (cat. no. H9523), chloroquine (cat. no. C6628), olopatadine hydrochloride (cat. no. O0391), resiniferatoxin (cat. no. R8756), capsaicin (cat. no. M2028), allyl isothiocyanate (AITC, cat. no. 377430), pertussis toxin (PTX, cat. no. P7208), and guanosine 5′-[β-thio]diphosphate trilithium salt (GDPβS, cat. no. G7637) were all purchased from Sigma-Aldrich (St. Louis, MO), whereas the selective and cell permeable FPR2 antagonist (PBP 10, cat. no. 4611) was obtained from Tocris (Minneapolis, MN). Mouse CGRP-targeting small interfering RNA (siRNA) (assay ID s233632) and non-targeting siRNA (cat. no. 4390844) were purchased from Thermo Fisher Scientific (Waltham, MA). *In vivo*-jetPEI® (cat. no. 201-10G) by Polyplus (New York, NY) was mixed with siRNA to increase the uptake of siRNA by DRGs.

### Acute itch and pain behaviors

Mice received intradermal microinjections of RvD3, phosphate-buffered saline (PBS), pruritogens or algogens intradermally (i.d.) in the cheek or in the paw 15,35. Testing pruritogen-induced acute itch behaviors, cheeks were shaved and mice were habituated to the recording chambers (15×25×10 cm) at least 2 days before the test. On the day of the experiment, mice were injected with 20 μL of RvD3 (10 ng, i.d.) or control PBS solution and placed in the recording chambers for 30 min. Mice were then removed from the chambers and given an intradermal injection in the cheek of 10 µL of the following pruritogens: histamine (100 µg), serotonin (4 µg), or chloroquine (100 µg), all diluted in saline containing 3.5% dimethyl sulfoxide. Immediately after the pruritogen injections, mice were placed again in the recording chambers and video recorded for 30 min. Spontaneous scratch responses were quantified by counting the scratching bouts of the hind paw to the shaved region injected with the pruritogen. Hind paw movements directed away from the injection site and grooming movements were not counted. To test algogen-induced acute pain behavior (nocifensive behavior), mice were intrathecally injected with 10 µL of RvD3 (10 ng, i.t.) or control PBS solution and placed in the recording chambers for 1 h. Then, mice were removed from the chambers and given an intraplantar injection of 20 µL in the paw of the following algogens: capsaicin (200 pmol) or AITC (200 nmol), both diluted in PBS. Immediately after the algogen injection, mice were placed again in the recording chambers and video recorded. Time spent on nocifensive behavior (flinching and licking) was recorded for 5 min. To assess a potential motor impairment induced by RvD3 (20 μL, 10 ng, i.d.), mice were put on a treadmill (rotarod) with accelerating speed, and the seconds spent before the first fall were recorded.

### Mouse model of psoriasis

We generated psoriasiform skin inflammation and itch in mice by application of imiquimod cream, as previously described 12,36. Briefly, mice received a daily topical application of 62.5 mg 5% IMQ cream on the shaved back skin (nape area of 2 × 2 cm) for 7 consecutive days, whereas control mice were treated similarly but with a control vehicle cream (Vaseline). Spontaneous itch, alloknesis, and skin inflammation were assessed daily or on day 2 and/or 7. For spontaneous itch, mice were habituated to the testing environment daily for at least two days before testing and itch assessed 20 to 22 hours after each topical application by videotaping the mice for 30 min. Spontaneous itch, alloknesis, and skin inflammation were assessed daily or on day 2 and/or 7. For spontaneous itch, mice were habituated to the test environment daily for at least two days before the test, and itch was assessed 20-22 h after each topical application by videotaping the mice for 30 min. Spontaneous itch was determined by the number of scratching bouts, and a bout was defined as one rapid back-and-forth hind paw motion directed toward and contacting the treated area, ending with licking or biting of the toes or placement of the hind paw on the floor. Hind paw movements directed away from the treated area (e.g., ear scratching) and grooming movements were not counted. For alloknesis, mice received five separate innocuous mechanical stimuli delivered using a von Frey filament (bending force: 0.07 g; Stoelting, Wood Dale, IL) to five randomly selected sites along the border of the cream application area. The presence or absence of a positive response (a hind limb scratching bout directed to the site of mechanical stimulation) was noted for each stimulus. The alloknesis score was the total number of positive responses elicited by the five stimuli (0-5). For skin inflammation, we evaluated erythema and scaling based on our and other similar studies 12,28,37. Briefly, erythema (red taint) and scaling (white plaques) were scored separately on a scale ranging from 0 to 4 according to the degree of severity: 0, absent; 1, mild; 2, moderate; 3, severe; 4, very severe. Skin inflammation was also assessed at the end of our behavioral analyses by hematoxylin and eosin (H&E) staining. Briefly, skin from the nape was fixed in 4% paraformaldehyde, embedded in tissue freezing medium, and cut into 20 µm-thick sections on a microtome. The sections were then stained with H&E, and epidermal thickness and number of immune cells were quantified as previously described 12,28.

### Peripheral sensory denervation

To examine the roles of TRPV1-expressing C-fibers in psoriasis, mice were injected once with capsaicin analogue resiniferatoxin (RTX, 50 µg/kg, subcutaneously) seven days prior to the imiquimod treatment, as described previously 38. Although denervated mice exhibited insensitivity to noxious heat stimuli, overall behavior qualitatively remained unaltered in RTX-treated mice.

### Quantitative real-time RT-PCR (qPCR)

Naïve mice were deeply anesthetized with isoflurane, perfused transcardially with PBS, and both DRG (C5-T3) and nape skin tissues removed immediately. Total RNA was extracted using the Direct-zol RNA MiniPrep kit (Zymo Research, Irvine, CA), the amount and quality of which were assessed by a SimpliNano UV-Vis Spectrophotometer (General Electric, Boston, MA), and then converted into cDNA using a high-capacity cDNA reverse transcription kit (cat. no. 4368814, Thermo Fisher Scientific). Specific primers for various cytokines and neuropeptides, as well as glyceraldehyde 3-phosphate dehydrogenase (GAPDH) were obtained from PrimerBank 39. Primer sequences are depicted in Supplementary Table S1. qPCR was performed on a QuantStudio 3 Real-Time PCR System (Thermo Fisher Scientific) using PowerUp SYBR Green Master Mix (cat. no. A25741, Thermo Fisher Scientific). All samples were analyzed at least in duplicate and normalized to GAPDH expression. The relative expression ratio per condition was calculated based on the method described by Pfaffl 40.

### Reverse-transcription polymerase chain reaction (RT-PCR)

cDNA was synthesized in the same way as described for qPCR. Samples were diluted 2:100 and used as a template for PCR experiments. The following primer pairs were used: mouse Fpr2 (forward, 5'-ACTGTGAGCCTGGCTAGGAA-3'; reverse, 5'-CATCAGTTTGAGCCCAGGAT-3'), mouse Gapdh (forward, 5'-TGAAGGTCGGTGTGAACGAATT-3'; reverse, 5'-GCTTTCTCCATGGTGGTGAAGA-3'), human GPR32 (forward, 5'-TTTGCCAGTAACTGCCTCCT-3'; reverse, 5'-TGTCAGAGTTGAACGCCAAG-3'), and human GAPDH (forward, 5'-ACCCAGAAGACTGTGGATGG-3'; reverse, 5'-TTCTAGACGGCAGGTCAGGT-3').

### Whole-cell patch clamp recordings in cultured mouse DRG neurons

DRGs from all spinal levels of 6-8-week-old mice were removed aseptically and incubated with collagenase (5 mg/mL, Roche, Basel, Switzerland) / dispase-II (1 mg/mL, Roche) at 37°C for 40 min, then digested with 2.5% trypsin (Invitrogen) for 7 min at 37°C, followed by 0.25% trypsin inhibitor (Sigma). Cells were mechanically dissociated with a flame polished Pasteur pipette in the presence of 0.05% DNAse I (Sigma). DRG cells were then plated on glass coverslips previously coated with a solution of 0.1 mg/mL poly-L-ornithine. DRG cells were grown in a Neurobasal medium (with 2% B27 supplement, Invitrogen), at 37 °C, with 5% CO2. DRG neurons were grown for 18 h before use 41,42. Whole-cell current-clamp recordings were performed at room temperature to measure currents with HEKA EPC10 (HEKA). The patch pipettes were pulled from borosilicate capillaries (Chase Scientific Glass Inc., Rockwood, CA, USA). When filled with the pipette solution, the resistance of the pipettes was 4 ~ 6 MΩ. The recording chamber was continuously perfused (2-3 mL/min). Series resistance was compensated for (> 80%), and leak subtraction was performed. The Pulse v8.30 software (HEKA) was used during experiments and analysis. The internal pipette solution was composed of (in mM): 140 KCl, 1 CaCl_2_, 2 MgCl_2_, 10 EGTA, 10 D-glucose and 10 HEPES adjusted to pH 7.3 with NaOH, osmolarity 295 - 300 mOsm. The extracellular solution contained (in mM): 140 NaCl, 5 KCl, 2 CaCl_2_, 1 MgCl_2_, 10 HEPES, and 10 D-glucose, adjusted to pH 7.3 with NaOH, osmolarity 300-310 mOsm. Voltage-clamp experiments were performed at a holding potential of -60 mV 15,41,42.

### CGRP release assay from skin explants and cultured neurons

Skin punch biopsies (10 mm) were collected from the nape of the murine psoriasis model and rapidly transferred into 24-well plates containing 1 mL of Dulbecco's modified Eagle medium. Explants were incubated at 38°C for 30 min. After incubation, the supernatant from the organ cultures was collected and assayed to measure CGRP concentrations with the CGRP EIA kit (cat. no. 589001, Cayman Chemical) according to the manufacturer`s instructions 43. The same kit was used to determine the amount of CGRP released into the media of cultured murine and human sensory neurons stimulated by capsaicin (1 µM, 30 min) with or without RvD3 (100 nM or 1 µg). The same procedure described in section 2.8. was used to obtain murine cultured sensory neurons, whereas human cultured sensory neurons were prepared as previously described 44.

### CGRP knockdown in mice

A total of 3 µg of siRNA targeting CGRP or non-targeting control siRNA were diluted in a 10 µl solution with 2.62 µL of *in vivo*- jetPEI® and 5% glucose. This solution was then injected intrathecally on days 0 and 5 after the first application of IMQ to investigate the role of CGRP in DRG tissues. A valid spinal puncture and intrathecal delivery of siRNA was confirmed by a reflexive tail flick after needle entry into the subarachnoid space, as previously described 12,15,45.

### Immunofluorescence

Mice were anesthetized terminally with isoflurane and perfused through the ascending aorta with saline, followed by 4% paraformaldehyde. DRGs (C5-T3) were removed and post-fixed in the same fixative overnight. DRG sections (12 μm) were cut in a cryostat and processed for immunofluorescence. Tissue sections were blocked with BlockAid^TM^ blocking solution (cat. no. B10710, Thermo Fisher Scientific) for 30 min and incubated overnight at 4°C with primary goat antibodies against CGRP (1:500; cat. no. ab36001, Abcam) and 4'-6-diamidino-2-phenylindole dihydrochloride (DAPI; 300 nmol/L; cat. no. D1306, Thermo Fisher Scientific). Sections were then incubated for 1 h at room temperature with secondary antibodies conjugated to Alexa Fluor 555 (1:500; cat. no. A-21432, Thermo Fisher Scientific). Immunostained tissues were examined under an Olympus fluorescence microscope (BX63), and images were captured with a high-resolution CCD Spot camera (cat. no. DP80, Olympus) and analyzed with CellSens (Olympus). All image acquisitions and intensity quantifications to compare samples from different experimental groups were performed under identical conditions, prepared with the same staining solutions and measured using identical display parameters. For immunoreactive intensity measurements, the intensities were quantified in three sections per mouse and five mice per group by individuals who were blinded to treatment conditions 46.

### Calcium imaging of cultured human DRG neurons

Human L4 and L5 DRGs were recovered from consented organ donors in collaboration with LifeCenter, Cincinnati and the University of Cincinnati Medical Center and prepared for calcium imaging as described previously 44. Briefly, human DRG neurons were incubated with Fura-2-AM (3 µg/mL; Thermo Fisher Scientific) for 40 min at 37°C in Neurobasal-A Media with B27, 2 mM GlutaMAX, 5% fetal bovine serum, and 1% penicillin-streptomycin (Gibco). During calcium imaging experiments, the extracellular solution contained (in mM) 130 NaCl, 5 KCl, 10 HEPES, 2 CaCl_2_, 1 MgCl_2_, and 30 D-(+)-glucose, pH 7.3. Neurons were illuminated through a 10× immersion objective on an Olympus BX51 microscope by 365 nm and 385 nm wavelength LEDs using a pE-4000 (CoolLED). Images were acquired on a Rolera Bolt CMOS camera connected to a PC running the Metafluor software (Molecular Devices). The neuronal identity was confirmed at the end of each experiment by a response to 50 mM KCl solution. All drugs were applied via bath perfusion at a flow rate of ~2 mL/min.

### Statistical analysis

Statistical analyses were performed with GraphPad Prism software (San Diego, CA), and all data were expressed as the mean ± standard error of the mean (SEM). Differences between groups were compared using Student's t-test (two groups), one-way analysis of variance (ANOVA) (multiple groups), or two-way ANOVA (multiple groups and time course), the latter two followed by Tukey's post hoc test. The criterion for statistical significance was *p* < 0.05 with *p < 0.05, **p < 0.01, and ***p < 0.001.

## Results

### RvD3 attenuates TRPV1-dependent acute itch and pain behaviors

**Figure 1:**
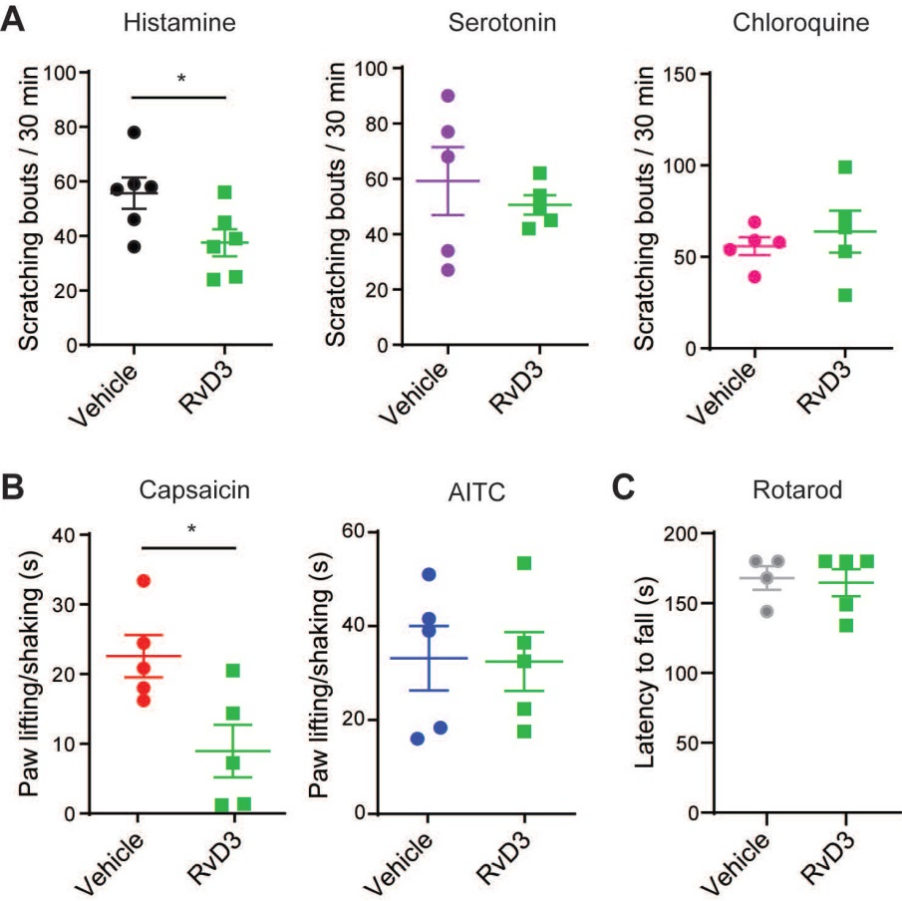
** RvD3 inhibits TRPV1-dependent acute itch and pain.** (**A**) Cheek-directed scratching bouts elicited in response to the intradermal injection of histamine, serotonin or chloroquine in mice treated with a vehicle control or RvD3 (n = 5-6 mice/group). (**B**) Paw lifting/shaking elicited in response to the intradermal injection of capsaicin or allyl isothiocyanate (AITC) in mice treated with a vehicle control and RvD3 (n = 5 mice/group). (**C**) Latency to fall assessed by rotarod in mice treated with a vehicle control or RvD3 (n = 4-5 mice/group). Statistical analysis: (A-C) two-tailed unpaired Student's t-test was used; data are depicted as mean ± SEM.; and *p < 0.05.

Although resolvins are canonically studied for their potent anti-inflammatory and pro-resolving actions in inflammatory diseases, they have recently emerged as novel analgesics modulating the activity of TRP channels in DRG neurons 20,47. Here, we first used different pruritogens and algogens to test whether RvD3 modulates specific TRP channel-dependent acute itch- and pain-like behaviors in mice. Intradermal injection (i.d.) of the pruritogens histamine (100 µg), serotonin (4 µg), or chloroquine (100 µg) in the cheek elicited acute itch-like behaviors quantified by the numbers of spontaneous scratching bouts directed to the injection site (Figure 1A). Additional administration of RvD3 (10 ng/20 µL, i.d.) lowered only the numbers of spontaneous scratching bouts elicited by histamine, which are well-known to be dependent on TRPV1 expression and activity 14. Accordingly, we found that intrathecal injection (i.t.) of RvD3 (10 ng/10 μL) significantly reduced pain-like behaviors (flinching/licking) in mice induced by intradermal injection of the TRPV1 agonist capsaicin (1 µg/20 µL), but not the TRPA1 agonist AITC (200 nmol/20 µL), in the hind paw (Figure 1B). To note, RvD3 (10 ng/20 µL, i.d.) did neither change basal motor skills assessed by rotarod (Figure 1C) nor mechanical and thermal sensitivity (Figure S1A-B). Thus, RvD3 significantly attenuated TRPV1-dependent acute itch- and pain-like behaviors without affecting normal motor and sensory functions.

### RvD3 reduces TRPV1 currents in DRG neurons via ALX/FPR2

**Figure 2:**
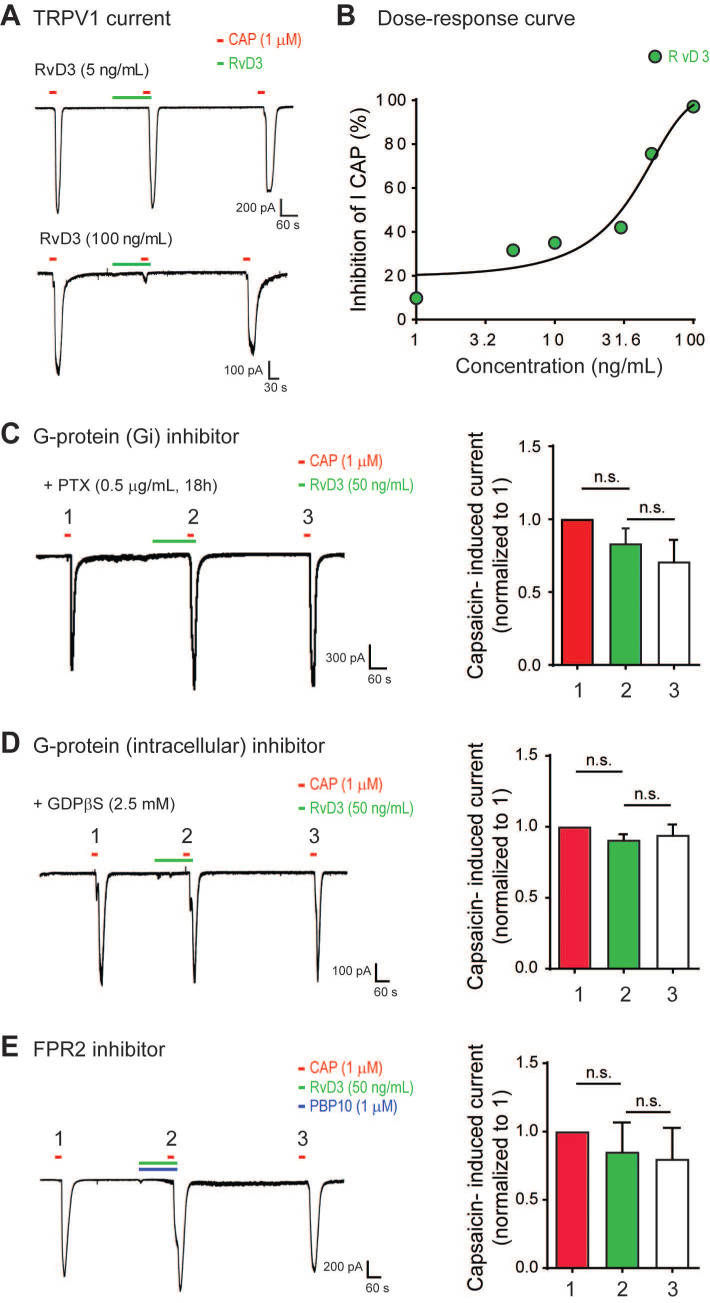
** RvD3 regulates TRPV1 currents in mouse DRG neurons via the G protein-coupled receptor ALX/FPR2.** (**A**) Representative capsaicin (CAP)-induced inward currents. Note the concentration-dependent inhibition of TRPV1 currents by RvD3. (**B**) Concentration-response curve showing the inhibition of TRPV1 currents by RvD3 (n = 4-6 neurons from 2 mice). (**C**) Pretreatment of DRG cultures with pertussis toxin (PTX) blocks the inhibitory effects of RvD3 on TRPV1 current (n = 5 neurons from 2 mice). (**D**) Intracellular perfusion with guanosine 5′-[β-thio]diphosphate (GDPβs; 8 min) blocks the inhibitory effects of RvD3 on TRPV1 current (n = 5 neurons from 2 mice). (**E**) The N-formyl peptide receptor 2 (FPR2) inhibitor PBP10 blocks the inhibitory effects of RvD3 on TRPV1 currents (n = 5 neurons from 2 mice). Statistical analysis: (C-E) one-way ANOVA followed by Tukey post-hoc test; data are depicted as mean ± SEM.; and n.s. = not significant.

To determine the neuronal and molecular mechanisms by which RvD3 regulates pain and itch, we used patch-clamp recordings to examine whether RvD3 directly modulates TRPV1 activity in small-diameter (<25 mm) dissociated mouse DRG neurons. Incubation of dissociated neurons with capsaicin (1 μM) induced a robust TRPV1 inward currents, which were dose-dependently inhibited by RvD3 (IC_50_ = 31.18 ng/mL) (Figure 2A-B). Resolvin actions are mediated by specific GPCRs, and in mice RvD3 exerts its activity via the N-formyl peptide receptor 2 also known as ALX/FPR2 29. To determine the involvement of GPCRs in the observed RvD3 effects, we pretreated DRG cultures with the selective G_αi_-coupled GPCR inhibitor pertussis toxin (PTX; 0.5 µg/mL) for 18 h. After PTX treatment, RvD3 was not able to inhibit TRPV1 currents (Figure 2C). We confirmed the dependence of the described RvD3 effects on G proteins by intracellular delivery of GDPβS (2.5 mM, 8 min) via the recording electrode, which also abolished the inhibition of capsaicin-induced TRPV1 currents by RvD3 administration (Figure 2D). To verify that RvD3 actions occurred via ALX/FPR2, we used the specific small-molecule inhibitor PBP10 (1 µM, 8 min), which completely blocked the RvD3-induced inhibition of TRPV1 currents (Figure 2E). Together, these data strongly suggest that RvD3 modulates TRPV1 activity in DRG neurons via the G protein-coupled receptor ALX/FPR2.

### RvD3 reverses psoriasiform spontaneous itch and alloknesis

**Figure 3:**
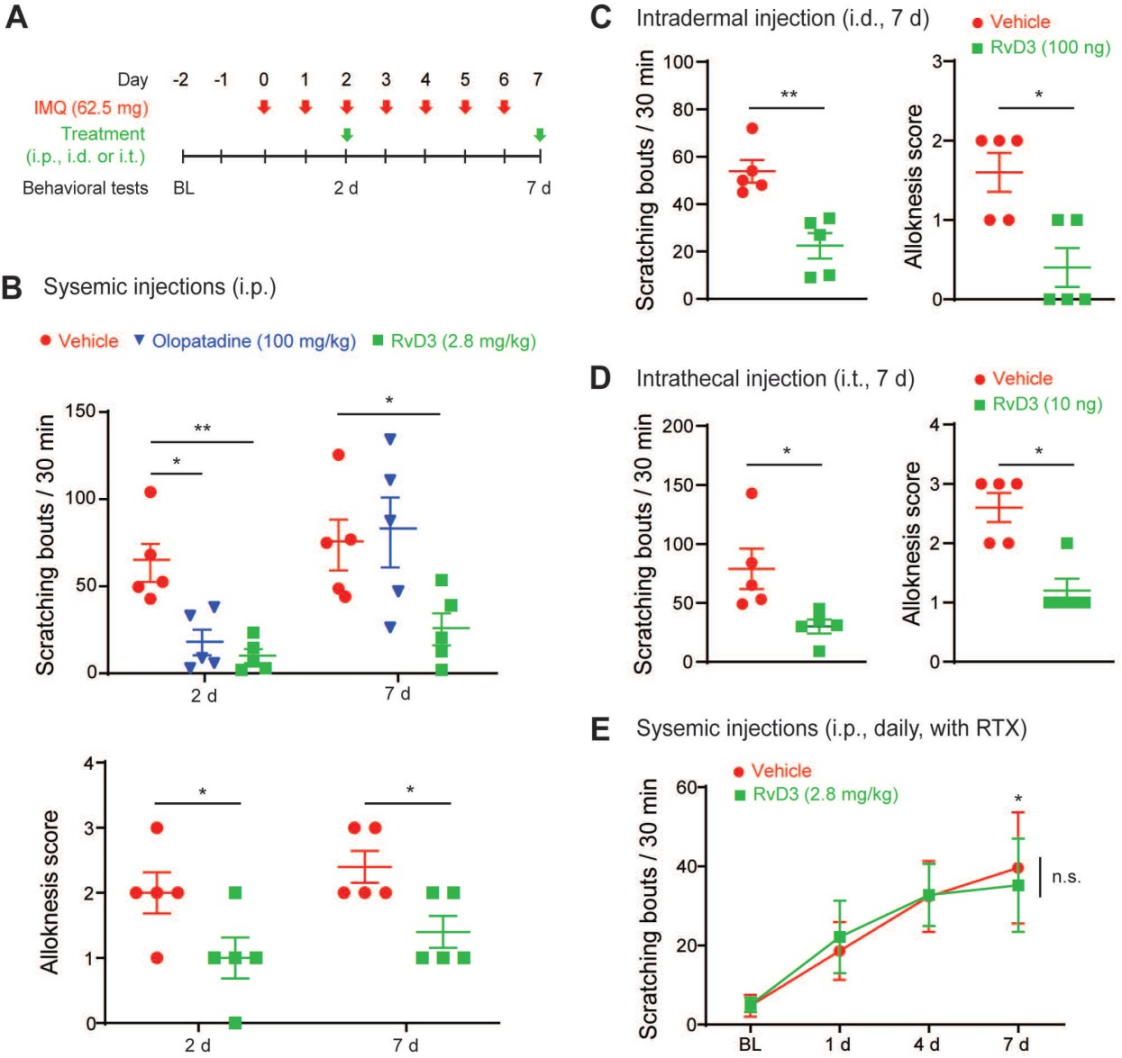
** Single administration of RvD3 reverses psoriasiform spontaneous scratching and alloknesis.** (**A**) Experimental schematic indicating the daily topical applications of imiquimod (IMQ) to the nape, as well as the times of the vehicle control, olopatadine, or RvD3 treatments and the behavioral tests. BL = Baseline (**B**) Numbers of spontaneous scratching bouts and alloknesis scores in mice intraperitoneally (i.p.) injected with different treatments at 2 and 7 days after the first IMQ application (n = 5 mice/group). (**C** and** D**) Numbers of spontaneous scratching bouts and alloknesis scores in mice with intradermal (i.d.) or intrathecal (i.t.) injection of control vehicle or RvD3 at 7 days after the first IMQ application (n = 5 mice/group). (**E**) Time courses of the numbers of spontaneous scratching bouts in mice treated with resiniferatoxin (RTX) and injected i.p. with vehicle control or RvD3 (2 injections, daily) (n = 5 mice/group). Statistical analysis: (B and E) two-way ANOVA followed by Bonferroni post-hoc test; (C-D) two-tailed unpaired Student's t-test; data are depicted as mean ± SEM.; and *p < 0.05, **p < 0.01. n.s. = non-significant.

Both TRPV1 and ALX/FPR2 are critical for the regulation of immunity and skin inflammation 1,48, with TRPV1 playing a major role in psoriasiform skin inflammation and potentially in chronic itch 11,18,19. Therefore, we asked whether RvD3 could reverse psoriasiform itch using a well-characterized preclinical mouse model of psoriasis. This model provokes itch and skin inflammation by repeated topical application of IMQ (Figure 3A) and closely resembles human psoriasis 11,36. We found that intraperitoneal injection (i.p.) of RvD3 (2.8 mg/kg) was able to reverse established spontaneous scratching 2 and 7 days after IMQ application, whereas injection of the commonly used antihistamine olopatadine significantly lowered the number of spontaneous scratching bouts only 2 days, but not 7 days, after IMQ application (Figure 3B). Alloknesis can be assessed by the number of positive responses to light touch stimuli and has been reported to be resistant to antihistaminic drugs 36. In our study, alloknesis was also reduced by systemic injection of RvD3 at both 2 and 7 days after IMQ application (Figure 3B). To further evaluate RvD3 as a potential therapeutic agent and understand the mechanisms associated with its antipruritic effects, we used different delivery routes and protocols. We found that IMQ-evoked spontaneous scratching and alloknesis were also attenuated by intradermal and, surprisingly, intrathecal injections of RvD3 (respectively: 100 ng/100 μl, i.d. and 10 ng/10 μl, i.t.) after 7 days (Fig 3C and D). Because intrathecal injection confines RvD3 to the nervous system, we hypothesized that RvD3 may attenuate psoriasiform itch solely through neuronal actions. To confirm this hypothesis, we examined the time course of the potential antipruritic effects of vehicle control and RvD3 (2.8 mg/kg, 2 injections, daily) in psoriatic mice pre-treated with resiniferatoxin (RTX) (Figure S2A), an ultrapotent TRPV1 agonist causing the loss of nociceptive fibers 11,49. Mice treated with RTX developed significant spontaneous scratching and skin inflammation (Figure 3E and Figure S2C-E), but not alloknesis (Figure S2B). Remarkably, the antipruritic effects of RvD3 on both spontaneous scratching and skin inflammation were abolished in these mice (Figure 3E and Figure S2C-E). Therefore, both electrophysiological and behavioral data suggest a distinct neuronal role of RvD3 in modulating TRPV1 activity, as well as in psoriasiform itch and skin inflammation.

### RvD3 dose-dependently prevents psoriasiform skin inflammation and chronic itch

**Figure 4:**
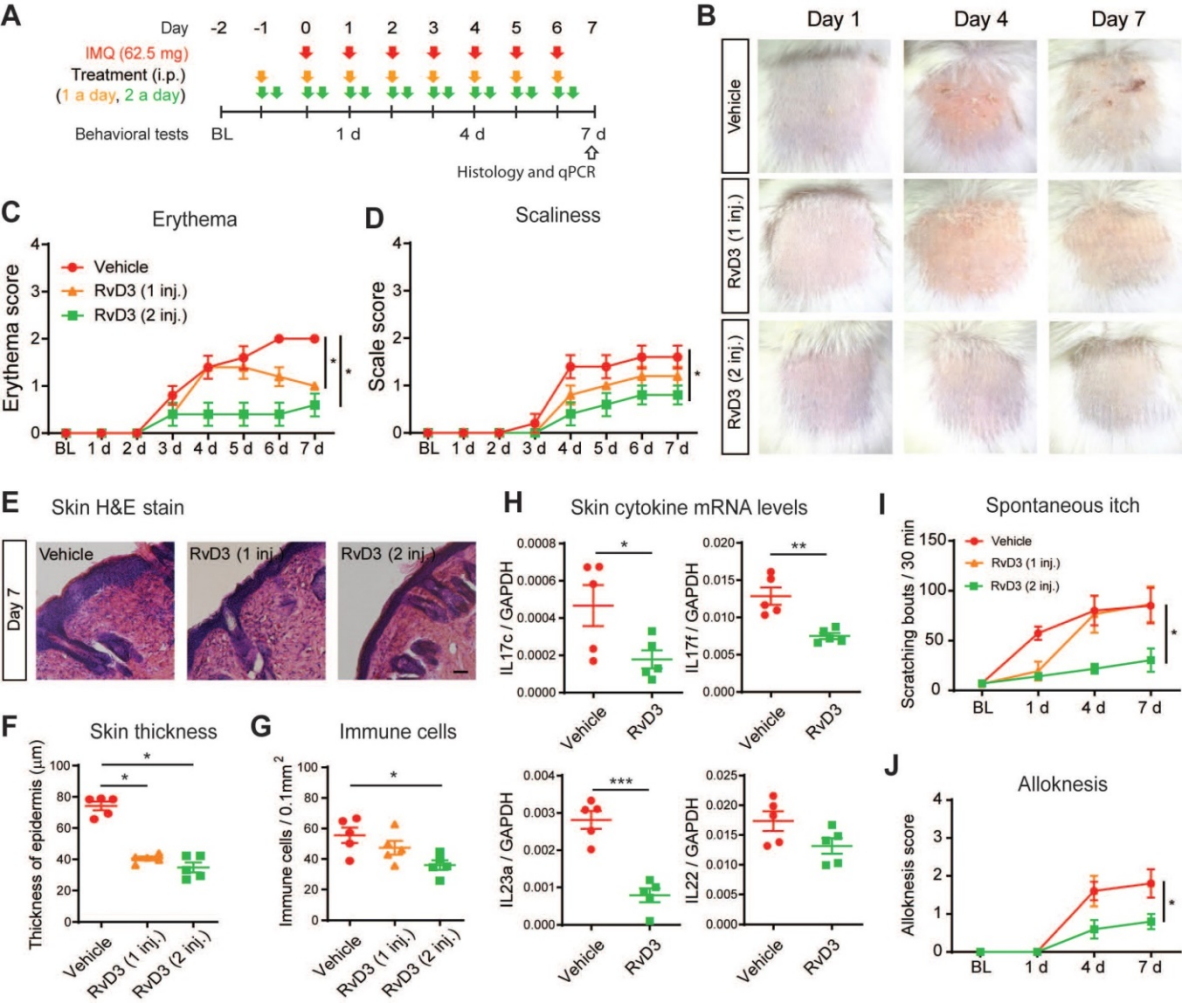
** Repeated administration of RvD3 prevents psoriasiform skin inflammation and itch.** (**A**) Experimental schematic indicating the daily topical applications with imiquimod (IMQ) to the nape, as well as the times of the vehicle control or RvD3 (1 and 2 daily doses, i.e., 1 inj. and 2 inj., respectively) treatments and the behavioral and biochemical tests. BL = Baseline (**B**) Representative images of skin lesions in the psoriasis model at different time points after intraperitoneal (i.p.) injections of vehicle control or RvD3 (1 inj. and 2 inj.). (**C** and** D**) Time course of erythema and scaliness in mice treated with i.p. injections of vehicle control or RvD3 (1 inj. or 2 inj.) (n = 5 mice/group). (**E-G**) Histopathology of skin tissues (Scale bar, 50 µm), epidermis thickness and immune cell infiltration in mice treated with i.p. injections of vehicle control or RvD3 (1 inj. and 2 inj.) at 7 days after first IMQ application (n = 5 mice/group). (**H**) Interleukin (IL)-17c, IL-17f, IL-22, and IL-23a mRNA expression levels at 7 days after the first IMQ application in skin tissues of mice treated with i.p. injections of vehicle control or RvD3 (2 inj.) (n = 5 mice/group). (**I** and **J**) Time courses of the numbers of spontaneous scratching bouts and alloknesis scores in mice treated with i.p. injections of vehicle control or RvD3 (1 inj. and 2 inj.) (n = 5 mice/group). Statistical analysis: (C, D, I, and J) two-way ANOVA followed by Bonferroni's post-hoc test; (F and G) one-way ANOVA followed by Tukey's post-hoc test; (H) two-tailed unpaired Student's t-test; data are depicted as mean ± SEM.; and *p < 0.05, **p < 0.01, ***p < 0.001.

We then asked whether systemic delivery RvD3 can dose-dependently prevent skin inflammation and chronic itch induced by repeated application of IMQ (Figure 4A). Erythema, scaliness and skin lesions are hallmarks of psoriasiform inflammation. We observed that mice treated with daily systemic administration of RvD3 (2.8 mg/kg, i.p., 1 or 2 injections daily) showed no skin lesions (Figure 4B) and a dose-dependent decrease in the development of both erythema and scaliness (Figure 4C-D). Epidermal thickening and infiltration of immune cells are also indicative of psoriasiform inflammation. Histologic examinations revealed that RvD3 treatment significantly attenuated the increase in thickness of the stratum corneum, as well as the cutaneous infiltration of immune cells (Figure 4E-G). Accordingly, RvD3 administration significantly decreased the transcriptional expression of the pro-inflammatory cytokines *Il17c*, *Il17f*, and *Il23a* in the skin (Figure 4H). Remarkably, RvD3 administration (2 injections daily) largely prevented the development of spontaneous itch (Figure 4I) and alloknesis (Figure 4J), suggesting the systemic delivery of RvD3 as a potential new therapeutic approach for the prevention of both psoriasiform skin inflammation and chronic itch.

### RvD3 decreases the expression and release of CGRP that controls psoriasiform skin inflammation and chronic itch

**Figure 5:**
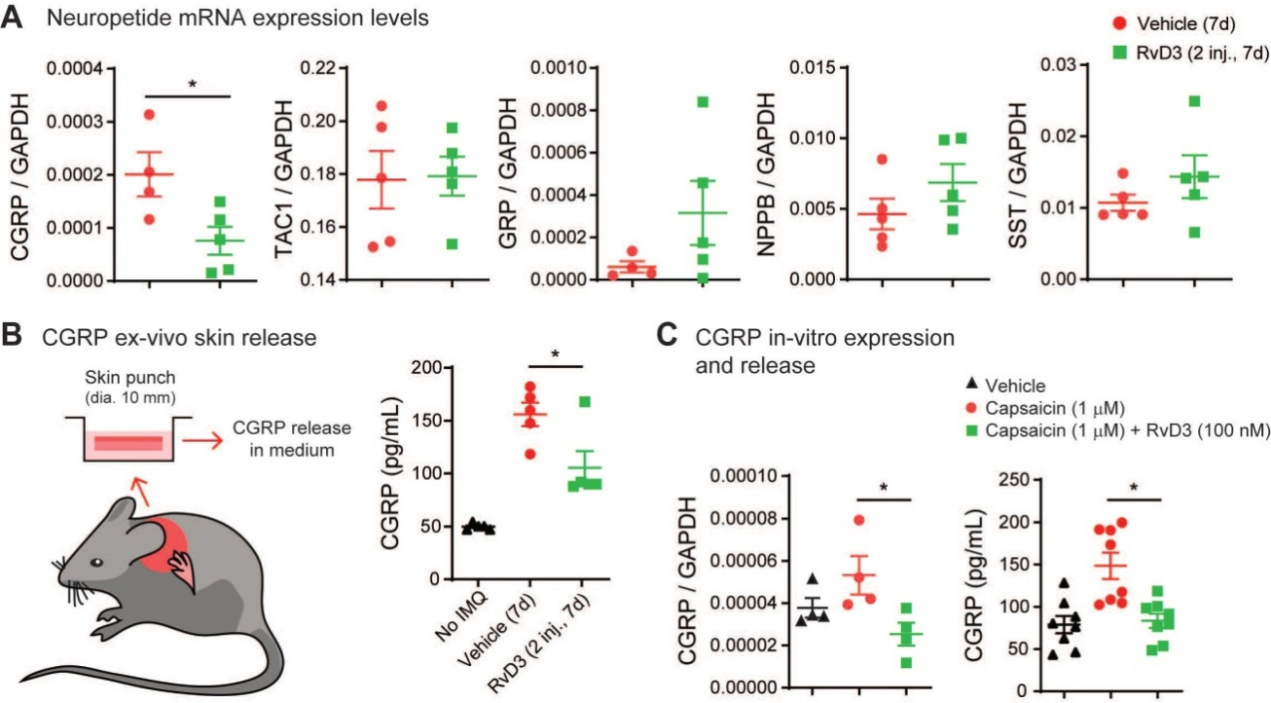
** RvD3 regulates CGRP expression and release in DRG neurons and psoriasiform skin tissue.** (**A**) Transcriptional expression levels assessed by qPCR of neuropeptides (NPPB, natriuretic peptide type B; SST, somatostatin; GRP, gastrin-releasing peptide; TAC1, tachykinin 1; CGRP, calcitonin gene-related peptide). To note, only CGRP levels in DRGs of psoriasis model mice are downregulated by repeated intraperitoneal (i.p.) injection of RvD3 (2 daily doses of 2.8 mg/kg, i.e., 2 inj.) at 7 days after the first imiquimod (IMQ) application (n = 4-5 mice/group). (**B**) Experimental design (skin explant) and CGRP protein release levels in *ex vivo* nape skin of punch biopsies at 7 days after the first IMQ application from mice treated with i.p. injections of vehicle control or RvD3 (2 inj.) (n = 5 mice/group). (**C**) CGRP mRNA expression and protein release levels in cultured DRG neurons incubated with vehicle control, capsaicin, or capsaicin with RvD3 (left, mRNA, n = 4; right, protein, n = 8). Statistical analysis: (A) two-tailed unpaired Student's t-test; (B and C) one-way ANOVA followed by Tukey's post-hoc test; data are depicted as mean ± SEM.; and *p < 0.05.

**Figure 6:**
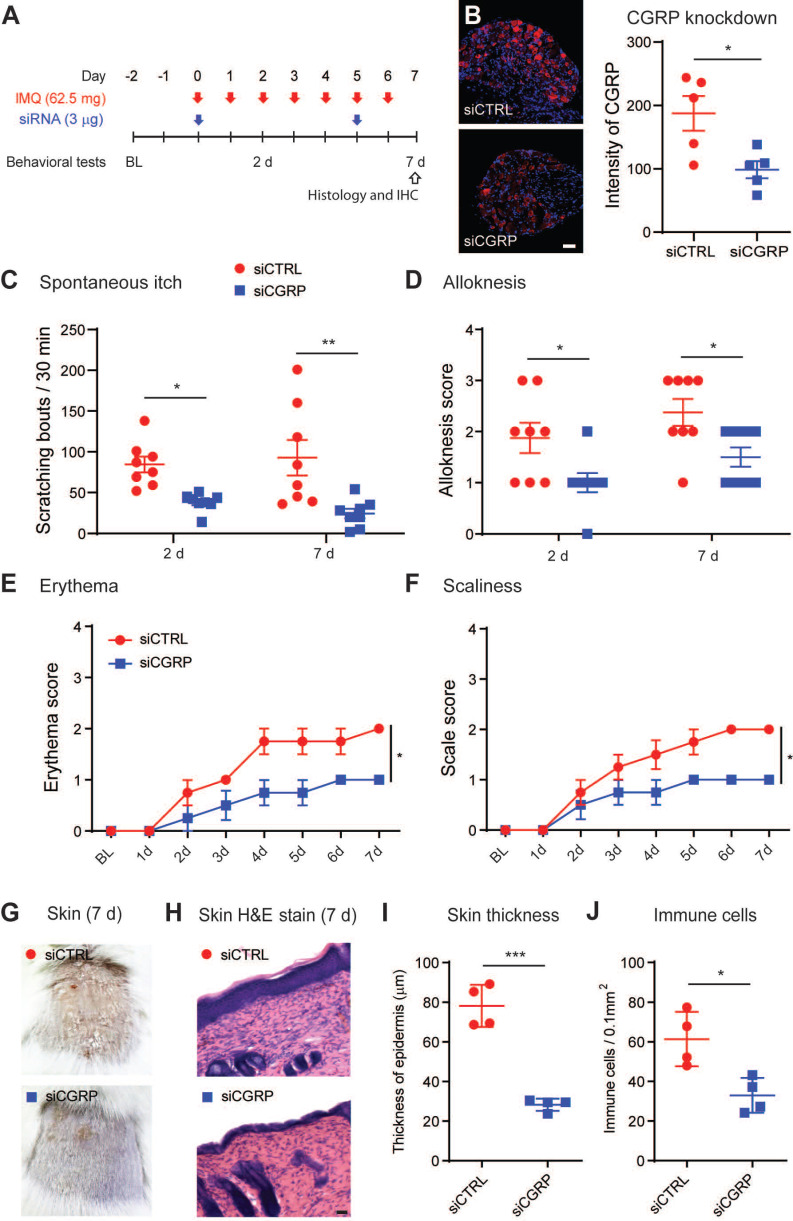
** CGRP knockdown in DRG tissue reduces psoriasiform itch and skin inflammation.** (**A**) Experimental schematic indicating the daily topical applications with imiquimod (IMQ) to the nape, as well as the times of the intrathecal siRNA deliveries (3 µg in 10 µL) and the behavioral tests. BL = Baseline (**B**) Representative immunofluorescent images of DRG tissues and quantification of the CGRP protein expression at 7 days after the initial application of IMQ in mice treated with a control siRNA (siCTRL) or siRNA targeting CGRP (siCGRP) (scale bar, 50 µm; n = 5 mice/group). Of note, siCGRP decreased CGRP expression by ~40%. (**C** and **D**) Numbers of spontaneous scratching bouts and alloknesis scores at 2 and 7 days after the first IMQ application in mice treated with siCTRL or siCGRP (n = 8 mice/group). **(E** and** F)**, Time course of erythema and scaliness in mice treated with siCTRL or siCGRP (n = 4 mice/group). (**G**) Representative images of skin lesions at 7 days after IMQ application in mice treated with siCTRL or siCGRP (**H-J**) Histopathology of skin tissues (scale bar, 50 µm), epidermal thickness and immune cell infiltration in mice treated with siCTRL and siCGRP (n = 5 mice/group). Statistical analysis: (B, I, and J) two-tailed unpaired Student's t-test; (C-F) two-way ANOVA followed by Bonferroni post-hoc test; data are depicted as mean ± SEM.; and *p < 0.05.

It has been suggested that peripheral nerve fibers control skin inflammation in a genetic mouse model of psoriasis via the increase and release of various neuropeptides 11,50. Thus, we investigated whether RvD3 attenuation of psoriasiform skin inflammation was associated with changes in expression levels of various neuropeptides. Our transcriptional analyses found that RvD3 (2.8 mg/kg, i.p., 2 injections daily) significantly decreased the transcriptional expression levels of CGRP in DRGs 7 days after IMQ application (Figure 5A), but not those of the pro-inflammatory substance P precursor *Tac1* or itch-related neuropeptides *Grp*, *Nppb* and *Sst* 51-53. We confirmed the regulation of CGRP protein release by RvD3 using an *ex vivo* organ culture of skin explants (Figure 5B). A significant increase in CGRP levels was observed in skin biopsies collected from mice 7 days after IMQ application, but this increase was significantly reduced in mice treated with RvD3 (2.8 mg/kg, i.p., 2 injections daily) (Figure 5B). Expression and release of CGRP were dependent on TRPV1-expressing neurons, as capsaicin (1 µM, 30 min) elicited in dissociated DRG neurons increases in CGRP transcription and release, which were significantly reduced by co-treatment with RvD3 (100 nM) (Figure 5C). Next, we asked whether the specific knockdown of CGRP in DRG neurons was sufficient to alleviate psoriasiform itch and skin inflammation. To this end, we used a well-characterized and previously described siRNA approach 12. Following two injections of siRNA (3 µg, i.t., on day 0 and 5) (Figure 6A), we observed a significant knockdown of the expression levels of CGRP protein in DRGs (~45%) without changes in CGRP mRNA in spinal cord (Figure S3A) in mice treated with a siRNA targeting CGRP (siCGRP) compared to mice treated with a control siRNA (siCTRL) (Figure 6B). Notably, knockdown of CGRP expression in DRG neurons resulted in a significant decrease in spontaneous itch and alloknesis 2 and 7 days after IMQ application (Figure 6C-D). Moreover, the progression of the skin inflammation was attenuated as indicated by the reduction in cutaneous erythema (Figure 6E) and scaliness (Figure 6F), as well as by the general improvement of the skin tissue (Figure 6G) with decreased thickness of the stratum corneum and reduced infiltration of immune cells (Figure 6H-J). Knockdown of CGRP expression in DRG neurons also resulted in the abolishment and significant decrease of the transcriptional expression levels of *Il17c* and *Il17f*, but no change was observed in the expression levels of *Il23a* in the skin (Figure S3B). These data suggest CGRP expression in DRGs as a major mediator, mainly through IL-17 signaling, of psoriasiform itch and skin inflammation.

### RvD3 reduces TRPV1 activity and CGRP release in human DRG neurons

**Figure 7:**
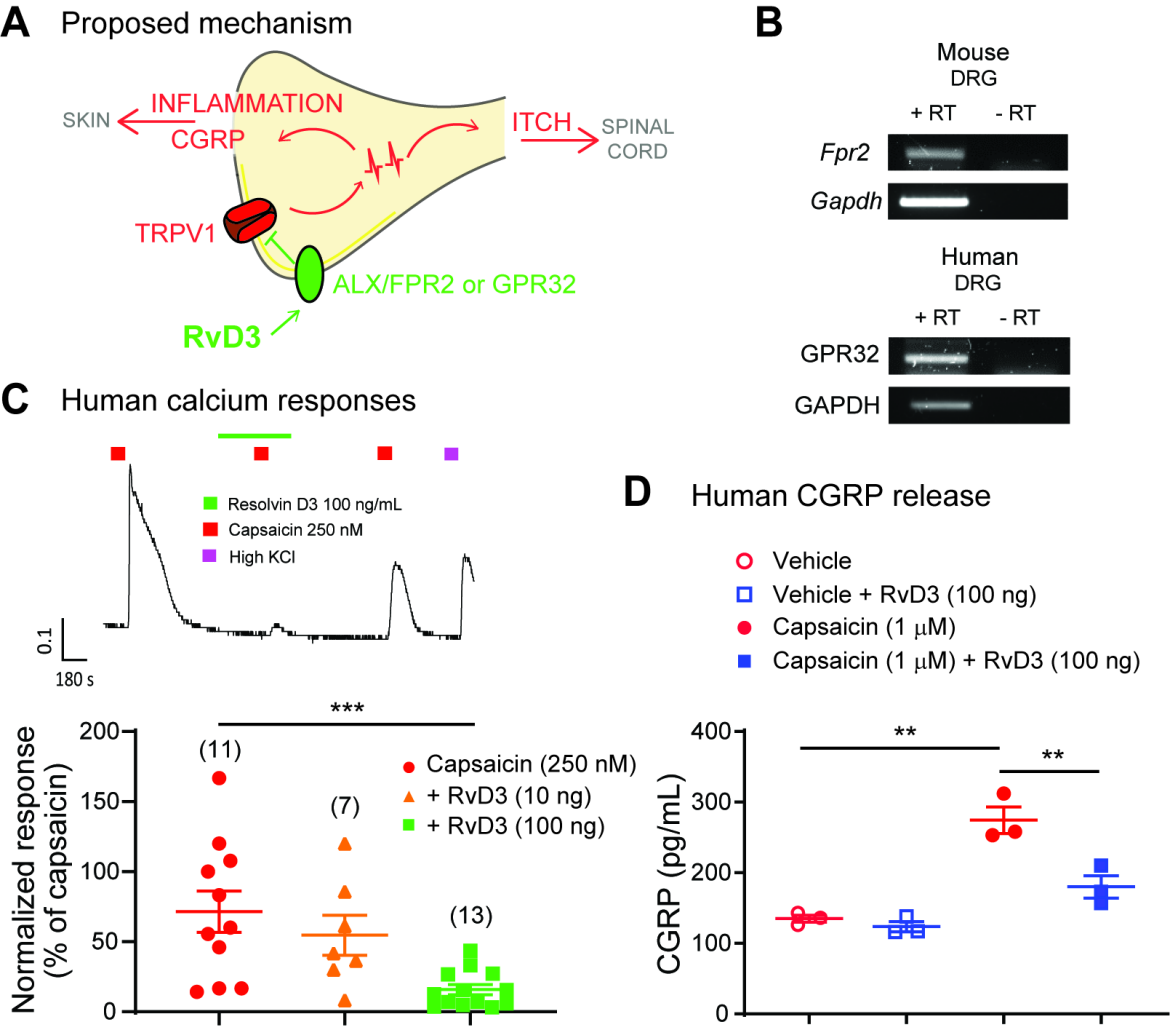
** RvD3 regulates TRPV1 activity and CGRP release in human DRG neurons.** (**A**) Schematic depicting the proposed mechanisms by which RvD3 reduces psoriasiform itch and skin inflammation. (**B**) Transcriptional expression of the RvD3 binding GPCR receptors in DRG tissues: mouse ALX/FPR2 and human GPR32. RT= reverse transcriptase. (**C**) Representative evoked calcium response and quantification in human DRG neurons after treatment with capsaicin and with capsaicin and RvD3. (**D**) CGRP protein release levels in cultured human DRG neurons incubated with vehicle control, vehicle and RvD3, capsaicin, or capsaicin and RvD3 (n = 3 cultures/group). Statistical analysis: (C and D) one-way ANOVA followed by Tukey's post-hoc test; data are depicted as mean ± SEM.; and **p < 0.01, ***p < 0.001.

Collectively our findings suggest that peripheral sensory neurons have a major impact in the development and progression of psoriasis in a preclinical animal model, and propose a molecular pathway by which RvD3 acts on the receptor ALX/FPR2 in DRG neurons controlling TRPV1 activity and CGRP release to attenuate both psoriasiform itch and skin inflammation in mice (Figure 7A). Is RvD3 relevant for a potential clinical treatment of psoriasis? Toward the human translation of our findings, we found that the ALX/FPR2 cognate human receptor GPR32 is expressed in human DRG tissue (Figure 7B), suggesting translation of the RvD3 molecular pathway and actions. Therefore, we tested whether RvD3 can block TRPV1 activity and CGRP release in human DRG neurons obtained from non-diseased donors. Calcium imaging of small-sized human DRG neurons (diameter < 60 μm) showed that capsaicin-induced responses were significantly suppressed by RvD3 treatment (100 ng/mL, Figure 7C). Similarly, the capsaicin-induced release of CGRP was significantly reduced by the same RvD3 treatment (Figure 7D). Therefore, we conclude that RvD3 has great translation potential as it also controls TRPV1 activity and CGRP release in human DRG neurons.

## Discussion

There is a growing appreciation that the activity of the nervous system plays an active role in various skin diseases, including psoriasis 5,7,8,11,12,50. However, most of the emerging therapies exclusively target immune signaling with various anti-inflammatory drugs 54,55 and monoclonal antibodies against single pro-inflammatory cytokines 56. Monoclonal antibodies have greatly advanced the clinical management of psoriasis, but not all patients are responsive to these treatments that can be expensive over prolonged periods, lead to unwanted effects such as immunosuppression, and occasionally even aggravate the disease 57,58. Lack of adequate treatments negatively influences the quality of patient life by deteriorating concentration, sleep, diet, and mood and promotes the misuse of drugs such as antidepressants and even opioids 59. Hence, novel therapeutics that can effectively and safely control psoriasis are still required. Here, we report that RvD3 administration targeting neuronal mechanisms underlying psoriasis can offer novel and safer therapeutic approaches.

Resolvins are derived from omega-3s and exhibit potent anti-inflammatory and pro-resolution actions in various animal models of inflammation, infection and pain 22,23. Resolvins have been shown to inhibit pain via effects on TRPA1 and TRPV1 channels 60 and we showed that RvD3 attenuated acute pain induced by the TRPV1 agonist capsaicin, suggesting potential analgesic actions. In contrast to other TRP channel inhibitors 21, no adverse effects have been reported for resolvins 23. However, further studies are required since omega-3s, from which resolvins are derived, are known to cause mild adverse effects such as nausea and diarrhea, and fishy taste in the mouth 61. TRP channels are emerging as essential drivers of itch, but the anti-pruritic actions of resolvins in animal models of itch remain unclear. We demonstrated herein that RvD3 reduced acute itch induced by histamine, but not that caused by serotonin or chloroquine. Histamine is one of the best-characterized itch mediators and binds to H1/H4 receptors on skin nerve terminals to elicit itch via activation of TRPV1 channels 14. By contrast, serotonin and chloroquine produce histamine-independent itch mainly via the respective TRPC4 and TRPA1 activation 15,16. Consistently, RvD3 reduced acute pain induced by the TRPV1-agonist capsaicin, but not the TRPA1-agonist AITC. RvD3 did alter neither basal thermal and tactile perceptions under normal conditions nor motor functions. Similar to other resolvins, RvD3 blocked capsaicin-induced TRPV1 currents in DRG neurons via activation of G_αi_-coupled GPCRs. It has been reported that RvD3 binds to the murine ALX/FPR2 receptor 29, and we confirmed that RvD3 blockade of capsaicin-induced TRPV1 currents was abolished when RvD3 was delivered in the presence of a specific ALX/FPR2 inhibitor. Together, these data suggest unique actions of RvD3 in resolving acute pain and itch via blockade of TRPV1 activity in DRG neurons.

Previous findings indicate that TRPV1-expressing neurons control cutaneous immune responses to pathogens 62,63, as well as skin inflammation in the IMQ animal model of psoriasis 11. Recent preclinical studies have shown that resolvins can also be beneficial in the very same animal model of psoriasis 27,28, but all these studies focused their attention only on skin inflammation disregarding the chronic itch associated with this condition. Yet chronic itch is possibly independent of the severity of the skin inflammation and is one of the most bothersome symptoms in psoriasis 64,65. We and others reported that antihistaminic drugs, which are widely used to treat acute itch, reduce spontaneous scratching only in the early phase (day 2), and not in the late phase (day 7) of psoriasis. Furthermore, they failed to inhibit alloknesis in either phase 36. By contrast, we found in the current study that a single systemic injection of RvD3 was sufficient to significantly inhibit both spontaneous scratching and alloknesis in early and late phases of IMQ- induced psoriasis. Interestingly, it has been reported that Merkel cells 66 and TLR5-expressing Aβ-LTMRs 67 in the periphery, as well as Unc3-expressing neurons in the spinal cord 68 are involved in the mechanisms of alloknesis. We found that RvD3 inhibitory effects are similar after local intradermal and intrathecal RvD3 injections, suggesting their independence from cells that are only present in skin or spinal cord tissues (i.e. Merkel cells and Unc3-expressing neurons). TLR5-expressing Aβ-LTMRs innervates both tissues, but this particular subpopulation of sensory neurons don not express TRPV1 69, which seems essential for RvD3 actions. Therefore, we suggest that RvD3 attenuates psoriasiform itch mostly via peripheral and spinal TRPV1-dependent neuronal mechanisms, similar to those previously proposed for inflammatory pain 70. Consistent with these neuronal mechanisms, RvD3 inhibition of spontaneous scratching and skin inflammation was abolished in mice treated with RTX, a chemical that ablates TRPV1-expressing neurons. Although our data highlight the neuronal mechanisms of RvD3 in psoriasis, we cannot rule out that RvD3 may also have additional immune mechanisms, as previously reported in preclinical models of other diseases 29,30,71.

Clinical studies suggest an imbalance between pro-resolution and pro-inflammatory mediators in the psoriatic skin 72 and propose foods and dietary supplements rich in omega-3s to be linked to a lower incidence of psoriasis, to a reduction in skin inflammation, and to an improvement in the quality of life of patients with psoriasis 61. In this regard, repeated systemic administration of RvD3 significantly prevented visual signs of psoriasis such as skin lesions, erythema, and scaliness. Consistent with therapeutic actions of RvD3 via TRPV1, we also demonstrated decreases in epidermal thickness and infiltration of immune cells as previously reported in preclinical studies using RTX-treated mice or TRPV1 knockout mice 11,18,19. This data correlates with significant reduction of pro-inflammatory cytokines IL-17c, IL-17f, and IL-23a, but not IL-22. IL-22 is predominantly produced by mast cells in patients with psoriasis 73, whereas the IL-17/IL-23 axis in dendritic and T-cells has been previously shown to be crucial for the development, progression and treatment of psoriasis 4,74. The observation that RvD3 administrations can prevent not only psoriasiform skin inflammation but also psoriasiform itch (i.e. spontaneous itch and alloknesis), supports a role for TRPV1-expressing neurons in psoriasis and the therapeutic importance of targeting neuroimmune mechanisms in this disorder.

It has been reported that TRPV1 expression in adult DRG neurons is mainly restricted to peptidergic nociceptors 75. Among various neuropeptides, we found that only the transcriptional expression of CGRP is downregulated in DRG tissues collected from mice treated with repeated RvD3 administration. Notably, similar downregulation of CGRP in DRG tissues was observed in RTX-treated mice 49. Furthermore, we confirmed this TRPV1-dependent regulation of CGRP by RvD3 using an *ex vivo* organ culture of skin explants from similarly treated mice and *in vivo* DRG neuronal cultures incubated with the TRPV1 agonist capsaicin to induce increases in CGRP expression and release. CGRP has also been implicated in skin inflammation and immunity 1, and in particular, CGRP levels in the plasma and in skin tissues were significantly higher in patients with psoriasis compared to healthy individuals 76,77. It has also been demonstrated that psoriasiform inflammation in denervated skin can be restored by intradermal delivery of CGRP 50, and we have recently suggested that inhibition of TRPC4 channels attenuates psoriasiform itch and inflammation potentially via decreased CGRP expression in DRG neurons 12. Although we propose CGRP regulation as the primary target by which RvD3 attenuates psoriasiform skin inflammation and itch, substance P has also been reported to restore psoriasiform skin inflammation in denervated skin by promoting the recruitment of immune cells 50. It is clear that additional neuropeptides that are enriched in nociceptors deserve further investigation, and may be critical to modulate inflammation and psoriasis 78.

Apart from its well-known roles in vasodilation and neuroimmune signaling, CGRP signaling and its inhibition have recently emerged as novel therapeutic approaches for migraine with several US Food and Drug Administration (FDA)-approved drugs currently on the market 79. Although several data suggest a critical role for CGRP in psoriasis 50,76,77,80, the therapeutic potential of its inhibition has only been investigated recently and remains controversial, showing minimal or no effects in psoriasiform skin inflammation and itch using global CGRP knockout mice or after the administration of CGRP receptor antagonist BIBN4096 49. These approaches have limitations that should be considered. Global knockout mice are prone to developmental compensatory mechanisms, and ablation of CGRP in the entire body eliminates the widely distributed and discrete functions of this neuropeptide in the peripheral and central nervous system 81, whereas systemic daily delivery of BIBN4096 may not reach the tissue of interest at required concentrations, or the drug may have a half-life too short to exert the desired actions. To overcome these limitations, we used the previously well-characterized intrathecal delivery of siRNA 15,45 to knockdown CGRP specifically in DRG neurons. Remarkably, we found that mice treated with siRNA targeting CGRP have significantly reduced psoriasiform itch and skin inflammation compared to mice treated with a control siRNA, indicating an important and local role of CGRP inhibition in psoriasis and a potential to repurpose the FDA-approved migraine drugs targeting CGRP signaling.

## Conclusions

In summary, we reported that RvD3 significantly alleviated psoriasiform itch and skin inflammation in the IMQ-animal model of psoriasis via activation of the ALX/FPR2 receptor, inhibition of TRPV1 activity, and reduction in CGRP release. But are these findings relevant for the treatment of psoriasis in patients? We believe they are because we found that: (1) the ALX/FPR2 cognate human receptor GPR32 was expressed in human DRG tissue, (2) RvD3 significantly reduced capsaicin-induced calcium responses in nociceptive human DRG neurons, and (3) RvD3 significantly decreased the capsaicin-induced release of CGRP in human DRG neurons. Together our findings suggest that RvD3 can control mouse and human DRG neuronal functions (i.e. TRPV1 activity and CGRP release) and significantly attenuate psoriasiform itch and skin inflammation. Given its neuroimmune actions and safety profile associated with dietary supplements, RvD3 and other drugs targeting its underlying neuronal signaling may represent a novel class of therapeutic agents for the clinical management of psoriasis.

## Supplementary Material

Supplementary figures and tables.
