# Supplementary material for: Resolvin D3 controls mouse and human TRPV1-positive neurons and preclinical progression of psoriasis
Source: Theranostics. 2020 Oct 26;10(26):12111–26. doi: 10.7150/thno.52135 (PMC7667671; doi:10.7150/thno.52135)
Supplement: Supplementary file 1 — Supplementary figures and tables. [file thnov10p12111s1.pdf]

Supplementary materials

Figures:

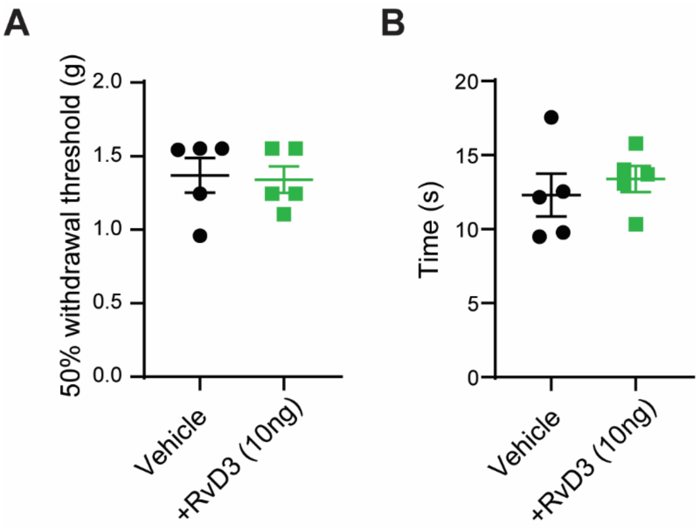

Figure S1: RvD3 does not alter mechanical and thermal sensitivity. (**A**) Mechanical sensitivity assessed by von Frey's test and (**B**) heat sensitivity assessed using the Hargreaves' in mice intradermally injected with RvD3 (10 ng) and a vehicle control (n = 5 mice/group). Statistical analysis: (A and B) two-tailed unpaired Student's t-test was used; data are depicted as mean ± SEM.

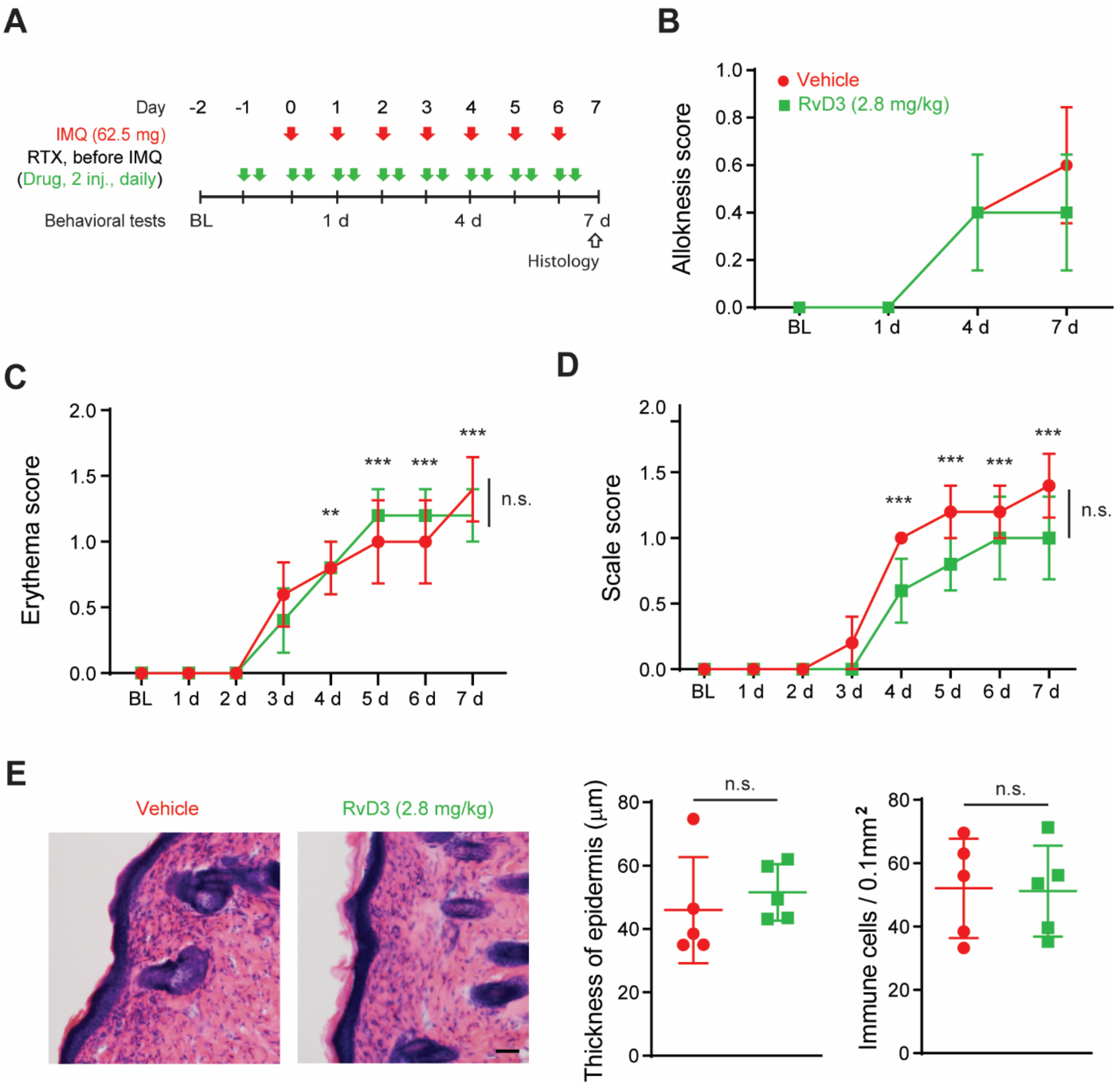

Figure S2: Time course of resiniferatoxin (RTX) and RvD3 treatments on psoriasiform itch and skin inflammation. (**A**) Experimental schematic indicating the daily topical applications of imiquimod (IMQ) to the nape of mice pre-treated with RTX, as well as the times of the vehicle control or RvD3 treatments and the behavioral tests. BL = Baseline (**B**) Time course of alloknesis scores, as well as (**C** and **D**) cutaneous erythema and scaliness in psoriatic mice (daily topical application of imiquimod cream), which were pre-treated with RTX and injected intraperitoneally with RvD3 (2 daily doses of 2.8 mg/kg, i.e. 2 inj.) or vehicle control (n = 5

mice/group). To note, spontaneous itch, erythema and scaliness significantly develop overtime in mice treated with RTX, but RvD3 treatments failed to show any additional effects compared to mice injected with a vehicle control. (**E**) Histopathology of skin tissues (Scale bar, 50μm), epidermis thickness and immune cell infiltration at 7 days after the first IMQ application in mice treated with RTX and injected intraperitoneally with RvD3 or vehicle control (n = 5 mice/group). Statistical analysis: (A-D) two-way ANOVA followed by Bonferroni post-hoc test; (E) two-tailed unpaired Student's t-test; data are depicted as mean ± SEM.; and $**p < 0.01$, $***p < 0.001$. n.s. = non-significant.

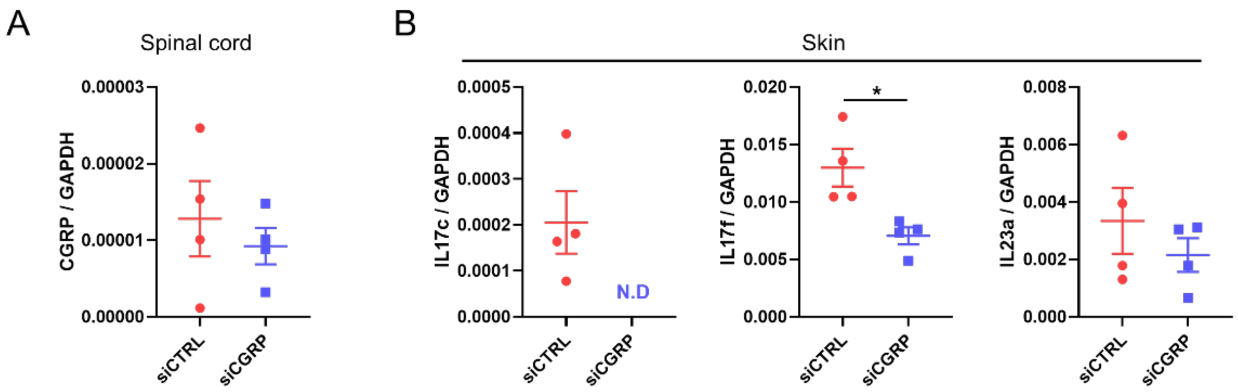

Figure S3: CGRP knockdown in DRG tissue reduces cutaneous levels of interleukin-17. (**A**) Spinal CGRP mRNA expression levels are unchanged, and (**B**) cutaneous interleukin (IL)-17c, IL-17f, and IL-23a mRNA expression levels at 7 days after the first imiquimod application in mice treated with intrathecal deliveries of siRNA (3 μg in 10 μL on day 0 and 5) against CGRP (siCGRP) or a control (siCTRL). Statistical analysis: (A and B) two-tailed unpaired Student's t-test was used; data are depicted as mean ± SEM.; and $*p < 0.05$.

Table:

Table S1. Primer sequences for qPCR

Target gene (product length)	Primer sequences	Accession number
IL17c (283bp)	ATGCTTGTGTCGTGGATG<br>GTGCCTGGAATGTCTGTC	NM_145834.3
IL17f (170bp)	TGCTACTGTTGATGTTGGGAC<br>AATGCCCTGGTTTTGGTTGAA	NM_145856.2
IL22 (124bp)	ATGAGTTTTTCCCTTATGGGGAC<br>GCTGGAAGTTGGACACCTCAA	NM_016971.2
IL23a (119bp)	TGGATACGGGGCACATTATTTT<br>CAGCAGCTCTCTCGGAATCTC	NM_031252.2
CGRP (102bp)	GAGGGCTCTAGCTTGGACAG<br>AAGGTGTGAAACTTGTTGAGGT	NM_007587.2
NPPB (100bp)	GAGGTCACTCCTATCCTCTGG<br>GCCATTTCCTCCGACTTTTCTC	NM_008726.5
SST (160bp)	ACCGGGAAACAGGAACTGG<br>TTGCTGGGTTCGAGTTGGC	NM_009215.1
GRP (166bp)	CTGTTGGCTCTGGTCCTCTG<br>CATACAGGGACGGGGATTCA	NM_175012.4
TAC1 (86bp)	ATTCCTTTGTTGGACTAATGGGC<br>ACGTCTTCTTTCGTAGTTCTGC	NM_001311060.1
